# Insights into the Protective Effects of Thymoquinone against Toxicities Induced by Chemotherapeutic Agents

**DOI:** 10.3390/molecules27010226

**Published:** 2021-12-30

**Authors:** Juveriya Farooq, Rokeya Sultana, Tahreen Taj, Syed Mohammed Basheeruddin Asdaq, Abdulkhaliq J. Alsalman, Mohammed Al Mohaini, Maitham A. Al Hawaj, Mehnaz Kamal, Saad Alghamdi, Mohd. Imran, Haleema Shahin, Ruheena Tabassum

**Affiliations:** 1 Department of Pharmacology, Yenepoya Pharmacy College and Research Centre, Yenepoya (Deemed to be University), Mangalore 575018, India; juveriyafarooq@gmail.com (J.F.); tahreentaj6414@gmail.com (T.T.); shahin@yenepoya.edu.in (H.S.); ruheenat@gmail.com (R.T.); 2 Department of Pharmacognosy, Yenepoya Pharmacy College and Research Centre, Yenepoya (Deemed to be University), Mangalore 575018, India; 3 Department of Pharmacy Practice, College of Pharmacy, AlMaarefa University, Riyadh 13713, Saudi Arabia; 4 Department of Clinical Pharmacy, Faculty of Pharmacy, Northern Border University, Arar 91911, Saudi Arabia; KALIQS@gmail.com; 5 Basic Sciences Department, College of Applied Medical Sciences, King Saud Bin Abdulaziz University for Health Sciences, Riyadh 31982, Saudi Arabia; mohainim@ksau-hs.edu.sa; 6 King Abdullah International Medical Research Center, Thuwal 31982, Saudi Arabia; 7 Department of Pharmacy Practice, College of Clinical Pharmacy, King Faisal University, Al-Ahsa 31982, Saudi Arabia; hawaj@kfu.edu.sa; 8 Department of Pharmaceutical Chemistry, College of Pharmacy, Prince Sattam Bin Abdulaziz University, Al-Kharj 11942, Saudi Arabia; mailtomehnaz@gmail.com; 9 Laboratory Medicine Department, Faculty of Applied Medical Sciences, Umm Al-Qura University, Makkah 21955, Saudi Arabia; Ssalghamdi@uqu.edu.sa; 10 Department of Pharmaceutical Chemistry, Faculty of Pharmacy, Northern Border University, Arar 91911, Saudi Arabia; imran.pchem@gmail.com

**Keywords:** thymoquinone, chemotherapy-induced toxicity, antioxidant, phytoconstituents, organ protection

## Abstract

The drugs used to treat cancer not only kill fast-growing cancer cells, but also kill or slow the growth of healthy cells, causing systemic toxicities that lead to altered functioning of normal cells. Most chemotherapeutic agents have serious toxicities associated with their use, necessitating extreme caution and attention. There is a growing interest in herbal remedies because of their pharmacological activities, minimal side effects, and low cost. Thymoquinone, a major component of the volatile oil of *Nigella sativa* Linn, also known as black cumin or black seeds, is commonly used in Middle Eastern countries as a condiment. It is also utilized for medicinal purposes and possesses antidiabetic, anti-cancer, anti-inflammatory, hepatoprotective, anti-microbial, immunomodulatory, and antioxidant properties. This review attempts to compile the published literature demonstrating thymoquinone’s protective effect against chemotherapeutic drug-induced toxicities.

## 1. Introduction

The National Cancer Institute defines cancer as a disease in which there is uncontrollable growth of the cell, spreading to other parts of the body [1]. According to the Global Burden of Disease, total cancer was found to be the second-most cause of deaths globally (9.36 to 10.6 million deaths) in 2019 [2]. Table 1 represents different types of cancer and total deaths from cancer in India [3]. Risk factors are classified as intrinsic risk factors and non-intrinsic risk factors. Intrinsic risk factors occur mostly because of errors in DNA replication and are always unmodifiable. Non-intrinsic risk factors are again divided into two types that are partially modifiable endogenous risk factors and modifiable exogenous risk factors. Partially modifiable endogenous risk factors include age, genetic susceptibility, DNA repair machinery, hormones, growth factors, and inflammation. Modifiable exogenous risk factors include exposure to radiation, chemical carcinogens (asbestos, Azodyes etc.), tumor causing viruses (Hepatitis C virus, Human papilloma virus, Epstein–Barr virus etc.), as well as lifestyle features including smoking, drinking alcohol, lack of exercise, imbalance in nutrition etc., [4]. All these risk factors contribute to the development of cancerous cells leading to disease progression, thus resulting in the presentation of multiple symptoms further deteriorating the quality of life [5]. Cancer mortality can be reduced if it is detected and treated at its earliest appearance [6]. It can also be prevented by adapting healthy lifestyle factors such as eliminating the use of tobacco, keeping a strong body mass index (BMI) and by limiting alcohol consumption [7]. Treatment approaches include surgery, radiotherapy, chemotherapy, hormone therapy, and molecular targeted therapy used alone or in combination with other therapies [8].

Chemotherapy involves the systemic application of chemicals or drugs to kill cancer cells. Different types of chemotherapeutic drugs are used to treat cancer and are classified based on their mechanism of action. Common drugs include: (1) alkylating agents which work by damaging the DNA and thus prevent the cell from reproducing. Examples include cisplatin, ifosfamide, chlorambucil, carmustine, melphalan, etc.; (2) antimetabolites which act as a substitute for the normal building blocks of RNA and DNA by interfering with their metabolism. Examples include: 5-fluorouracil, capecitabine, 6-mercaptopurine, cytarabine, gemcitabine, methotrexate, etc.; (3) antitumor antibiotics that act by binding with DNA and prevent the synthesis of enzymes involved in DNA replication. Examples include doxorubicin, daunorubicin, epirubicin, bleomycin etc.; (4) topoisomerase inhibitors also known as plant alkaloids, which work by inhibiting topoisomerases enzymes (I & II) involved in separating the strands of DNA during replication and transcription process. Examples include: irinotecan, topotecan, etoposide, teniposide, etc.; (5) mitotic inhibitors which are plant alkaloids responsible for inhibiting mitosis and cell division by disrupting microtubules essential for cell reproduction. Examples include docetaxel, paclitaxel, vincristine, vinblastine etc.; (6) corticosteroids which bind to cytoplasmic receptors thus inhibiting DNA synthesis and relieve the side effects caused due to other chemotherapeutic agents. Examples include prednisone, methylprednisolone, and dexamethasone [9].

However, the disadvantage of using chemotherapy is that it affects the healthy cells leading to altered functioning of normal cells. Most of the chemotherapeutic agents have serious toxicities associated with their use. Some of the common toxicities involved are nephrotoxicity, cardiotoxicity, hepatotoxicity, pulmonary toxicity, hematological, gastrointestinal, skin and hair follicle toxicity, nervous system toxicity, local toxicity, urinary tract toxicity, gonadal toxicity, etc. [10]. Techniques for reducing chemotherapy-induced toxicity include reductions in dosage, the usage of alternate medications, growth factors, and cytoprotective agents [11]. If the use of chemotherapy causes life threatening toxicity, it is essential to remove the offending agent making the therapy incomplete. For this reason, studies are being focused on use of safer therapeutic compounds.

Phytochemicals are a class of naturally occurring compounds that are used as medicinal agents in the treatment of a variety of diseases [12]. This paper provides an overview of thymoquinone’s protective function in lowering chemotherapeutic drug-induced toxicities. An extensive literature survey was conducted using common databases such as PubMed, Scopus, Google Scholar, Science Direct, Springer link, research gate, SAGE journals and other related published manuscripts.

## 2. Phytochemicals

Phytochemicals are biologically active plant-derived compounds which have been extracted from various sources including fruits for example, apples and grapes, vegetables such as onion and broccoli, spices including turmeric, herbal teas like green tea etc. [13,14]. Alkaloids, glycosides, flavanoids, phenolics, saponins, tannins, and terpenes are some of the phytochemical chemicals found in plants [15]. The majority of them have a wide range of pharmacological actions and are well tolerated [16]. Phytochemicals have been shown to decrease risk of a wide variety of illnesses such as autoimmune disease [17], cardiovascular disease [18], neuro-degenerative disorder [19], cancer [20], aging [21], obesity [22] and many more. As chemotherapy is associated with adverse effects, the addition of some phytochemicals to the regimen has the potential to improve their efficacy and alleviate their toxicities. The mechanism involved in reducing chemotherapeutic drug-induced toxicities is likely due to the anti-oxidative and anti-inflammatory properties. These constituents effectively scavenge reactive oxygen species, preventing oxidative stress and attenuating inflammation [23].

## 3. Thymoquinone (TQ)

**Figure 1:**
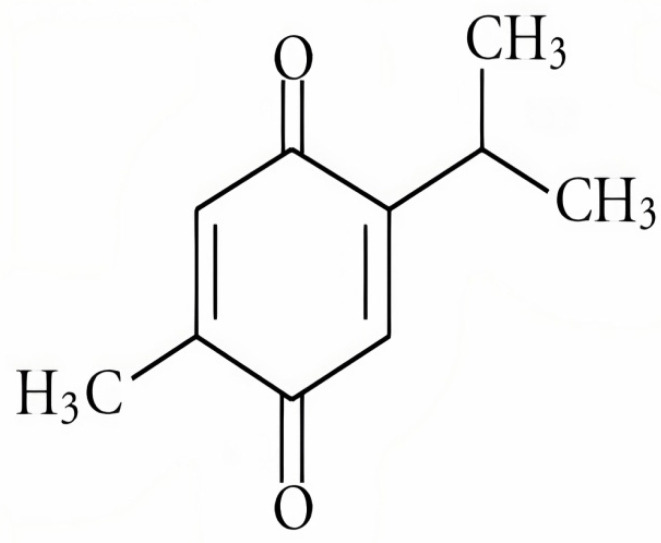
The chemical structure of thymoquinone [24].

**Figure 2:**
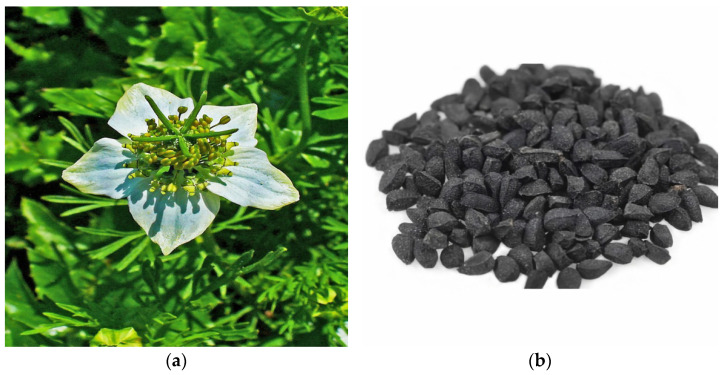
*Nigella sativa* (**a**)flower (**b**) seeds [26,27].

Thymoquinone (2-Isopropl-5-methyl benzo-1,4-quinone) (Figure 1) [24] is an active component of the volatile oil of *Nigella sativa* (black seeds) (Figure 2) [25,26,27]. Other than the Ranunculaceae family (*N. sativa*), this compound has been detected in other families such as Lamiaceae (*Modernadidyma*, *M. menthifolia*, etc.), Asteraceae (*Eupatorium cannabinum*), and Cupressaceae (*Juniperus communis*) [28]. It has a broad range of pharmacological actions involving anti-oxidative, anti-inflammatory, immunomodulatory, anti-cancer, anti-microbial, hepatoprotective, hypoglycemic and antidiabetic, gastroprotective, neuroprotective, cardioprotective, nephroprotective, hypolipidemic, and anti-histaminic effects. It also showed a protective effect on reproductive system, respiratory system, and bone related disorders [29]. TQ is generally safe to use but toxicity has been observed at higher doses (LD_50_, 2.5 g/kg). In a study conducted by Abukhader, Wistar Albino rats administered with 30 mg/kg and 40 mg/kg of TQ showed signs of toxicity such as lethargy, abdominal swelling, piloerection, irritability, and weight loss. It was found that 22.5 mg/kg and 15 mg/kg are the maximum tolerated doses (MTD) through the intraperitoneal route (I.P) in male rats and female rats, respectively. When administered orally at doses of 500 mg/kg and 300 mg/kg, toxicity signs like dyspnea, abdominal distension, weight loss, diarrhea, and hypoactivity were observed. The MTD through oral route (P.O) for both male as well as female rats were reported as 250 mg/kg. MTD is the highest safe dose that can be administered without showing signs of toxicity [30].

### 3.1. Role of Thymoquinone in Cancer

There are many preclinical studies (both in vivo and in vitro) which demonstrate the effect of TQ, given alone or in combination with other chemotherapeutic agents. It is known to target various processes of the cancer model such as cell cycle progression, proliferation, apoptosis, angiogenesis, migration, invasion, and metastasis of tumors. It also prevents oxidative damage and inhibits inflammatory responses [29]. TQ showed therapeutic efficacy against a wide range of cancer including ovarian cancer [31,32], breast cancer [33,34], pancreatic cancer [35], lung cancer [36,37], fibrosarcoma [38], neuroblastoma [39], osteosarcoma [40], myeloma [41], oral cancer [42], colon cancer [43,44], prostate cancer [45], squamous cell carcinoma [46], gastric cancer [47], leukemia [48], cervical cancer [49], liver cancer [50,51], and skin cancer [52]. In addition to its chemotherapeutic effects, it also diminishes the toxic side effects caused by chemotherapeutic agents.

### 3.2. Effect of TQ against ChemotherapyInduced Organ Toxicity

Toxicity is a major concern for chemotherapeutic drugs. Some of the most widely used chemotherapeutic drugs possess organ toxicity which leads to further economic burden and decreased quality of life. Isolated phytoconstituents are equipotent to synthetic analogues and have lesser side effects. Table 2 shows the findings of previous studies of thymoquinone in reducing toxicity of chemotherapeutic agents.

#### 3.2.1. Protective Effect against Cardiotoxicity

Many studies have demonstrated that TQ has a cardioprotective effect against chemotherapeutic drug-induced cardiotoxicity. Doxorubicin is an antitumor antibiotic used in the treatment of various types of cancer but its use is restricted due to the dose limiting cardiotoxicity. Alam et al. [23] conducted a study which demonstrated that serum enzyme markers of cardiotoxicity such as AST, ALT, CK-MB, and LDH in doxorubicin treated groups were elevated. MDA, a final product of lipid peroxidation, which act as an indicator for oxidative stress was increased and antioxidant enzymes such as CAT, SOD, GPx, GR, GST and GSH were decreased in doxorubicin treated groups. Oxidative stress occurs due to increase in reactive oxygen species generation and/or weakened antioxidant defense mechanism. Upon TQ treatment in mice (10 mg/kg, 20 mg/kg p.o), elevated levels were restored to normal. They also suggested that restoration may be due to the high antioxidant property of thymoquinone in the myocardium, thus minimizing the damage to the cardiac muscle fibers caused by doxorubicin. Co-treatment with TQ also restored the elevated levels of inflammatory cytokine (IL2) in cardiac tissue. These results were in accordance with other studies on doxorubicin-induced cardiotoxicity [53,70,71]. Karabulut et al. [71] performed a histopathological examination which revealed myocardial fibril disorganization in DOX-treated groups which was almost normally organized after treatment with TQ (10 mg/kg i.p). ANP and NT-proBNP values were determined, revealing that a doxorubicin dose of 15 mg/kg, i.p resulted in cardiac dysfunction leading to heart failure. These values were decreased by administration of thymoquinone before and after DOX-treatment. TQ supplementation also prevented cyclophosphamide-induced cardiotoxicity by restoring the abnormal levels of serum cardiac markers, lipid peroxidation, antioxidant enzymes and inflammatory mediators [54]. Cisplatin treatment caused myocardial tissue necrosis and apoptosis as evidenced by histopathological examination, which was diminished after TQ administration [55].

#### 3.2.2. Protective Effect against Hepatotoxicity

Tamoxifen is commonly recommended for treatment of patients with breast cancer [72]. It has the unusual side effect of producing hepatotoxicity, making long-term use challenging. Treatment with tamoxifen to rats resulted in significant increase in ALT, ALP, AST, LDH, γGT and total bilirubin. Histopathological changes included swelling of interstitial tissues and inflammation of Von Kuppfer cells with atrophy. There was a significant increase in lipid peroxidation level, inflammatory marker (TNF-α) and a significant decrease in antioxidant levels. All these changes ameliorated after administration of TQ [56]. It also showed hepatoprotective activity against cisplatin, cyclophosphamide and methotrexate toxicity with similar improvements in hepatic serum markers, lipid peroxidation, inflammatory markers, and antioxidant levels [57,58,59]. As a defence mechanism, inducible nitric oxide synthase (iNOs) is known to produce significant levels of nitric oxide; however, the excess production may cause damage to the liver. El-Sheikh et al. [59] performed immunohistochemical staining of the liver in methotrexate-treated rats and it was observed that iNOs expression upregulated, which confirmed nitrosative stress. Nitric oxide levels were also increased in cisplatin-induced hepatotoxicity [57]. These effects were reversed after treatment with TQ, suggesting that it has an antinitrosative effect. All these data show that TQ has hepatoprotective activity and can be used as an adjuvant in treating hepatotoxicity caused by chemotherapeutic drugs.

#### 3.2.3. Protective Effect against Nephrotoxicity

Literature survey revealed the protective effect of thymoquinone against nephrotoxicity in rats when given in combination with doxorubicin, cisplatin and ifosfamide. Badary et al. [58] demonstrated that doxorubicin treatment produced renal changes as manifested by hypoproteinemia, proteinuria, albuminuria, hyperlipidemia, hypoalbuminemia. They also evaluated oxidative stress as examined by lipid peroxide generation, non-protein sulfhydryl (NPSH) concentration and catalase (CAT) activity in renal tissue. These changes were improved after TQ administration to nephritic rats. A study was conducted on both rats as well as mice by Badary et al. [59] revealing that the rats were more sensitive to cisplatin-induced nephrotoxicity than mice. It was found that serum urea and serum creatinine levels were elevated with decreased creatinine clearance, increased urinary volume and renal tubular damage was also observed in the cisplatin treated group. Similar changes were also observed in ifosfamide-induced nephrotoxicity [63]. Upon TQ administration, the abnormal levels were restored to normal. TQ showed protection against doxorubicin-induced nephrotoxicity by improving lipid peroxidation, antioxidant levels, nitrosative stress markers as well as inflammatory markers [62].

#### 3.2.4. Protective Effect against Intestinal Toxicity

Methotrexate is known to cause serious intestinal toxicity. A study has demonstrated the mechanism by which TQ shows protection against methotrexate-induced intestinal toxicity. It acts by decreasing oxidative and nitrosative stress markers in intestinal mucosa, decreasing expression of inflammatory markers (TNF-α, NF-κB and COX-2) and by inhibiting apoptosis [64]. Another study by Shahid et al. [65] estimated the effect of long-term cisplatin use in rat intestines and the role of TQ in preventing toxicity. It was observed that repeated doses of cisplatin increased MDA levels, decreased GSH and TSH levels, as well as other antioxidant enzymes such as SOD, GSH-Px, CAT, GST, GR and TR in intestinal mucosa. Glutathione peroxidase, glutathione reductase, and glutathione-s-transferase are the main GSH-dependent antioxidant enzymes. Histopathology of brush border mucosa (BBM) revealed morphological changes such as congestion, swelling of villi, alteration in the contour and increased lymphocytic infiltration in the lamina propria associated with reduction in the crypt/villus ratio. It was further noticed that there was a significant reduction in enzyme markers of brush border mucosa which are sucrase, ALP, GGTase, and LAP. They also observed alterations in enzymes involved in carbohydrate metabolism. These abnormalities in both methotrexate and cisplatin were restored to normal upon TQ administration.

#### 3.2.5. Protective Effect against Urotoxicity

Cyclophosphamide-induced hemorrhagic cystitis is the dose limiting toxicity causing damage to urothelium of bladder mucosa. However, administration of TQ has shown protective activity against it by increasing antioxidant enzymes, decreasing lipid peroxidation, reducing levels of inflammatory markers, and maintaining the structure and morphology of the urinary bladder by increasing Nrf2 expression. Oxidative stress causes the level of Nrf2 to decrease [66].

#### 3.2.6. Protective Effect against Ototoxicity

Sagit et al. [65] demonstrated that cisplatin-induced ototoxicity in rats is caused due to an increase in ABR thresholds and a decrease in DPOAE responses. The intensity of sound at which a brain response initially appears is determined by ABR analysis [73]. DPOAEs are emission of sounds in response to two tons of different frequencies played at the same time [74]. Accumulation of cisplatin in cochlear tissues generates excessive free radicals and decreases antioxidant enzymes which results in cochlear injury or cell death. TQ administration preserved the changes produced by cisplatin treatment [67].

#### 3.2.7. Protective Effect against Testicular Injury

Gokce et al. [68] identified rise in TAC level and myeloperoxidase activity in methotrexate treated groups. An increase in TAC is a result of compensation to oxidative stress generated while increased myeloperoxidase activity indicates neutrophil accumulation leading to oxidative testicular damage. The seminiferous epithelium was severely disrupted, reducing the diameter of the seminiferous tubules and affecting the spermatogenetic cell lines. It also caused interstitial space dilation and edema in mice with cell size reduction and cytoplasmic swelling. TQ exerted its protective effect by restoring the abnormalities caused produced by methotrexate treatment.

#### 3.2.8. Protective Effect against Pulmonary Toxicity

A study was conducted by Suddek et al. [69] to demonstrate acute pulmonary toxicity induced by cyclophosphamide. They observed increase in MDA levels, SOD (as a compensatory mechanism), total protein, serum LDH, TNF-α and a decrease in GSH. Histopathological examination of the lung revealed congestion, damage, swelling of interalveolar septum, neutrophilic, and macrophages infiltration. TQ supplementation resulted in reversal of the changes produced by Cyclophosphamide.

## 4. Role of Thymoquinone against Chemotherapy Induced Oxidative Stress

Reactive oxygen species (ROS) are produced as a result of various cellular processes involving endogenous and exogenous pathways, constituting oxidative stress [75]. Endogenous pathways include oxidative phosphorylation, bacterial invasion, and inflammation; whereas exogenous pathway includes xenobiotics, radiation, and pollution. ROS are implicated in the anticancer mechanism of chemotherapy which further causes adverse effects. In other words, it has been established that chemotherapeutic agents have the potential to induce oxidative stress during the treatment [76]. Conklin [77] reported that among anticancer drugs, oxidative stress was observed to be the highest in theanthracycline class of drugs followed by platinum compounds, alkylating agents, epipodophyllotoxins and camptothecins but was lower in taxanes, antimetabolites, and vinca alkaloids. Hence the generation of ROS following the administration of chemotherapeutic agents decreases their efficacy. Inorder to prevent the side effects and to enhance responsiveness to therapy, antioxidants are recommended. SOD and CAT are antioxidant enzymes which are primarily responsible for destroying reactive oxygen metabolites. Glutathione enzymes (GSH, GPx, GR, GST) have an important role in providing secondary defense against oxidative damage caused by the generation of ROS [78]. Previous studies (refer Table 2) described how the supplementation of thymoquinone to experimental animals treated with chemotherapeutic agents increased antioxidant enzyme activity and thus showed some protection against organ damage.

## 5. New Trends and Directions of Research Related to TQ

Due to poor pharmacokinetic characteristics of TQ, its use in humans is limited as it is rapidly eliminated and slowly absorbed. To enhance bioavailability, several researchers developed nanoformulations of TQ which showed marked improvement in pharmacokinetic properties. In 2017, the United States government registered a clinical trial which evaluated the chemopreventive effect of TQ on oral potentially malignant lesions. Currently there are three completed trials and two ongoing trials registered for thymoquinone [79].However, there are no registered clinical trials on role of thymoquinone in reducing the toxicity of anticancer drugs. There are sufficient in vitro and in vivo studies describing the potential of TQ in reducing chemotherapeutic drug-induced toxicity, hence undertaking future clinical investigation in this particular area would be interesting in order to improve the efficacy of chemotherapy by diminishing the adverse effects produced during treatment.

## 6. Conclusions

TQ’s influence on cancer, metabolic syndrome, drug-induced toxicity in general, antioxidant, anti-inflammatory, immunomodulatory, antibacterial activity, and many other topics has been discussed in previous review publications. This is the first study to look at the effect of TQ on anti-cancer drug-induced toxicity. As a result, we emphasize that TQ has excellent organoprotective properties and might be utilized in conjunction with chemotherapy to minimize toxicity. The studies above show that TQ protects against the side effects of some of the most regularly used chemotherapy drugs. However, more research on diverse types of chemotherapeutic drugs is needed to have a better knowledge of their protective effects on several other physiological parameters.

## Figures and Tables

**Table 1:** Cancer types and total deaths from cancer in India, in 2019.

Sl. No.	Types of Cancer	Total Deaths in Percentage
1	Lung cancer	0.95%
2	Breast cancer	0.89%
3	Stomach cancer	0.87%
4	Colorectal cancer	0.84%
5	Lip and oral cavity cancer	0.70%
6	Other malignant neoplasm	0.61%
7	Cervical cancer	0.48%
8	Esophageal cancer	0.41%
9	Leukemia	0.36%
10	Pancreatic cancer	0.36%
11	Prostate cancer	0.34%
12	Liver cancer	0.33%
13	Larynx cancer	0.32%
14	Ovarian cancer	0.24%
15	Bladder cancer	0.14%

**Table 2:** Effect of thymoquinone against chemotherapy-induced toxicities.

Drug Induced Toxicity	Experimental Model	TQ Dose	Effect of TQ
Doxorubicin induced cardiotoxicity [23,53]	Swiss albino mice/adult male albino rats.	10 mg/kg p.o and 20 mg/kg, body weight p.o in swiss albino mice. 10 mg/kg/day i.p in albino rats.	↓ blood serum markers (AST, ALT, LDH, CK-MB, and CPK). ↓ lipid peroxidation levels (MDA). ↑ GSH. ↑ antioxidant enzymes (CAT, SOD, GPx, GR, and GST). ↓ inflammatory Cytokine (IL2).
Cyclophosphamide induced cardiotoxicity [54]	Adult male albino wistar rats	50 mg/L in drinking water (calculated dose of TQ- 4 mg/kg/day)	↓ CK-MB, LDH, Serum cholesterol, TG, urea, creatinine. ↑ ATP production. ↓ TBARS and NO(x). ↑ GSH, SOD, GPx, and CAT. ↓ proinflammatory mediator (TNF-α).
Cisplatin induced cardiotoxicity [55]	Adult male albino wistar rats	40 mg/kg/day i.p	Restored myocardial damage as observed by histopathological changes. ↑ expression of Bcl-2(anti-apoptotic protein) in myocardial fibers, indicating decreased apoptotic cardiomyocytes.
Tamoxifen induced hepatotoxicity [56]	Adult female Sprague-Dawley rats	50 mg/kg, body weight p.o	↓ serum enzymes of liver such as AST, ALT, γGT, LDH and ALP and total bilirubin. ↓ liver Lipid peroxidation level and TNF-α. ↑ GSH, SOD. Improved histopathological changes (edema of interstitial tissues and inflammation with decreased size of von kuppfer cells).
Cisplatin induced hepatotoxicity [57]	Male Albino wistar rats	500 mg/kg/day p.o	↓ serum hepatic biomarkers.(ALT, ALP, AST, γGGT, TB, LDH and ↑ serum albumin levels). ↑ GSH-px, SOD, GST, GSH, CAT activities. ↓ MDA formation. ↓ iNOs, TNFα and IL-1β, NF-Κb-P65 activation.
Cyclophosphamide induced toxicity [58]	Male Albino wistar rats	10 mg/kg, intragastric injection	↓ AST, ALT, ALP, γ-GT and CPK levels. ↓ elevated levels of urea, creatinine and bilirubin. ↓ TG, cholesterol, and LDL levels. ↑ GSH and decreased MDA levels.
Methotrexate induced hepato-renal toxicity [59]	Adult male albino wistar rats	10 mg/kg/day p.o	Improved renal and hepatic biomarkers (↓ elevated levels of BUN, creatinine, ALT and AST). ↑ GSH, CAT. ↓ renal and hepatic MDA, NO, and TNF-α levels. ↓ expression of iNOs in both kidney and liver. Improved renal and hepatic histology ↓ NF-*κ*B, COX-2 and caspase 3 expressions in kidney and liver.
Doxorubicin induced hyperlipidemic nephropathy [60]	Male albino wistar rats	10 mg/kg/day p.o	↓ serum urea. ↑ serum proteins and albumin. ↓ Urinary protein, albumin and NAG excretions. ↓ TG and TC in blood and renal tissue. ↓ Renal TBARS levels ↑ renal NPSH content and CAT activity.
Cisplatin induced nephrotoxicity [61]	Swiss albino mice, Wistar albino rats	8 mg/kg/day for mice, 4 mg/kg/day for rats p.o	↓ serum urea, serum creatinine, and urine volume in both mice and rats. ↑ creatinine clearance. Improved histopathological changes in rats (less degenerative damage and decreased loss of the tubular epithelium).
Doxorubicin induced nephrotoxicity [62]	Male Sprague–Dawley rats	50 mg/kg/day p.o	↓ creatinine, BUN and albuminuria. ↓ lipid peroxidation in renal cells. ↑ SOD and GST. Restored Nrf2 mRNA and Nrf2 binding activity in kidney. Attenuated renal NOX-4 levels. ↓ IL6 and TNF-α and ↑ IL-10. Improved renal histopathology (almost normal renal tubules and glomeruli).
Ifosfamide induced nephrotoxicity [63]	Male wistar albino rats	50 mg/L p.o	↓ urea and creatinine levels in the blood. ↑ serum phosphate, albumin content and creatinine clearance. ↓ fractional and total excretion of sodium, potassium, phosphate, glucose and organic acids. ↑ GSH, GST. ↓ Lipid peroxides.
Methotrexate induced intestinal toxicity [64]	Adult male rats	10 mg/kg/day, gastric gavage	Improved intestinal histology (mild shortening of villi present). ↑ intestinal GSH, CAT and ↓ MDA levels. ↓ rise in total nitrite/nitrate levels and iNOS intestinal expression. ↓ TNF-α and ↓ expression of NF-κB and COX-2 in rat intestine. Reversed the up regulation of caspase 3.
Cisplatin induced intestinal toxicity [65]	Adult male Wistar rats	1.5 mg/kg body weight, p.o	↓ MDA levels and ↑ GSH; total SH levels. ↑ activities of SOD, GSH-Px, CAT, GST, GR and TR and in intestinal mucosa. ↑ ALP, GGTase, LAP, sucrose and decreased ACPase activity. Significantly altered glucose metabolism enzymes in the mucosal homogenate (↓ LDH, HK, ME and increased MDH, G6Pase, FBPase, G6PDH activity) Preserved intestinal histopathology (protected against the damage caused by cisplatin on morphology of intestine)
Cyclophosphamide induced hemorrhagic Cystitis [66]	Male Balb/c mice	5, 10 and 20 mg/kg, i.p	TQ (20 mg/kg) showed complete protection of bladder tissues against inflammatory changes when compared with its low and medium dose. Reversed the Nrf2 suppression and most prominent Nrf2 protein expression was seen in the group receiving 20 mg/kg of TQ. ↓ TNF-α, IL-1β and IL-6 levels in a dose related manner. ↓ MDA level and significantly ↑ GSH, SOD, CAT levels in bladder tissue homogenates.
Cisplatin induced ototoxicity [67]	Female Sprague-Dawley rats	40 mg/kg/day i.p	TQ treatment preserved DPOAE responses and ABR thresholds.
Methotrexate induced testicular injury [68]	Male C57BL/6 mice	10 mg/kg/day i.p	↓ TAC values and myeloperoxidase activity. Upon microscopic examination of testes, treatment with TQ revealed almost normal seminiferous tubule morphology
Cyclophosphamide induced pulmonary toxicity [69]	Male Sprague-Dawley rats	100 mg/kg/day, p.o.	↓ serum total protein, TNF-a, TBARS level and SOD activity. Improved histopathological changes (no intralobular necrosis or substantial inflammatory infiltration, indicating minimal lung damage.)

## Abbreviation

ABR	Auditory brainstem response
ALT	Alanine aminotransferase
ANP	Atrial natriuretic peptide
AST	Aspartate aminotransferase
ATP	Adenosine triphosphate
BUN	Blood urea nitrogen
CAT	Catalase
CK-MB	Creatine kinase myocardial band
CPK	Creatine phosphokinase
DOX	Doxorubicin
DPOE	Distortion product otoacoustic emissions
FBPase	Fructose 1.6-bisphosphatase
G6Pase	Glucose 6-phosphatase
G6PDH	Glucose 6-phosphate dehydrogenase
GGTase	γ-Glutamyl transferase
GPx	Glutathione peroxidase
GR	Glutathione reductase
GSH	Reduced glutathione
GST	Glutathione S-transferease
HK	Hexokinase
IL-2	Interleukin-2
IL-6	Interleukin-6
IL-10	Interleukin-10
IL-18	Interleukin-18
iNOs	Inducible nitric oxide synthase
I.P	Intraperitoneally
LAP	Leucine aminopeptidase
LDH	Lactate dehydrogenase
MDA	Malondialdehyde
MDH	Malate dehydrogenase
ME	Malic enzyme
NAG	*N*-acetyl-b-D-glucosaminidase
NO(x)	Total nitrate/nitrite
NPSH	Non-protein sulfhydryl
Nrf2	Nuclear factor erythroid 2-related factor 2
P.O	Per os
ProBNP	Pro-B type natriuretic peptide
SH	Sulfhydryl
SOD	Superoxide dismutase
TAC	Total antioxidant capacity
TAS	Tissue anti-oxidant status
TC	Total cholesterol
TG	Triglycerides
TOS	Tissue oxidant status
TQ	Thymoquinone
TR	Thioredoxin reductase
